# Tannic acid-mediated synthesis of flower-like mesoporous MnO_2_ nanostructures as T_1_–T_2_ dual-modal MRI contrast agents and dual-enzyme mimetic agents

**DOI:** 10.1038/s41598-023-41598-0

**Published:** 2023-09-05

**Authors:** Farzaneh Sorouri, Elham Gholibegloo, Tohid Mortezazadeh, Sahar Kiani, Alireza Foroumadi, Loghman Firoozpour, Mehdi Khoobi

**Affiliations:** 1 Department of Radiopharmacy, Faculty of Pharmacy, Tehran University of Medical Sciences, Tehran, Iran; 2 Department of Brain and Cognitive Sciences, Cell Science Research Center, Royan Institute for Stem Cell Biology and Technology, ACECR, Tehran, Iran; 3 Department of Medical Physics, School of Medicine, Tabriz University of Medical Sciences, Tabriz, Iran; 4 Department of Medicinal Chemistry, Faculty of Pharmacy, Tehran University of Medical Sciences, Tehran, Iran; 5 Biomaterials Group, Pharmaceutical Sciences Research Center, The Institute of Pharmaceutical Sciences (TIPS), Tehran University of Medical Sciences, Tehran, 1417614411 Iran

**Keywords:** Cancer imaging, Chemistry, Nanoscience and technology

## Abstract

This study introduces a simple method for preparing a new generation of MnO_2_ nanomaterials (MNMs) using tannic acid as a template. Two shapes of MnO_2_ NMs, flower-like M1-MnO_2_ and near-spherical M2-MnO_2_, were prepared and compared as dual-active nanozymes and contrast agents in magnetic resonance imaging (MRI). Various parameters, including the crystallinity, morphology, magnetic saturation (M_s_), surface functionality, surface area, and porosity of the MNMs were investigated. Flower-like M1-MnO_2_ NMs were biocompatible and exhibited pH-sensitive oxidase and peroxidase mimetic activity, more potent than near-spherical M2-MnO_2_. Furthermore, the signal intensity and r_1_ relaxivity strongly depended on the crystallinity, morphology, pore size, and specific surface area of the synthesized MNMs. Our findings suggest that flower-like M1-MnO_2_ NM with acceptable dual-enzyme mimetic (oxidase-like and peroxidase-like) and T_1_ MRI contrast activities could be employed as a promising theranostic system for future purposes.

## Introduction

Diagnostic science, as an interdisciplinary field, has long been known to understand the components, processes, dynamics, and treatment of a disease at the biochemical and molecular levels^1–3^. Magnetic resonance imaging (MRI) has emerged as a non-invasive molecular imaging method for the early detection, diagnosis, and visualization of diseases. Unlike X-ray computed tomography (CT) and positron emission tomography (PET), MRI does not utilize ionizing radiation^4,5^. However, contrast agents (CAs)-free MRI is a double-edged approach for indicating soft tissues. The long relaxation time of water protons (H^+^) results in images with poor contrast and low sensitivity. Therefore, CAs can be used to differentiate the normal and abnormal tissues, leading to more accurate diagnosis of pathologies^6^. Paramagnetic and superparamagnetic metals are mainly utilized as CAs in MRI, generating high-resolution information regardin spatial and temporal characters of typical and malignant cellular processes. Most of the currently used T_1_- or T_2_-MRI CAs are based on gadolinium (Gd), manganese (Mn), and magnetite nanoparticles (Fe_3_O_4_ NPs)^7–10^. To date, Gd has been the predominant lanthanide used as a T_1_-positive CA. Owing to the seven unpaired electrons, Gd^+3^ possesses a high net magnetic moment. However, due to the potential toxicity and short blood circulation times, Food and Drug Administration (FDA) has limited clinical applications of Gd-based CAs. Nephrogenic systemic fibrosis in patients with severe renal failure is also another important drawback of the Gd-based CAs^7,11^. A few iron-based nanoparticles (NPs) have been approved by FDA as T_2_-negative CAs. Despite some interesting features of Fe_3_O_4_ NPs such as low toxicity and high stability, application of these NPs is restricted due to their intrinsic dark signals and magnetization artifacts which lead to the bleeding defects and thus misdiagnosis^9^. To overcome these drawbacks, Mn-based CAs have gained considerable attention because of their biocompatibility and positive contrast enhancement. Mn, as a non-lanthanide metal resembling Ca^2+^, is a natural cellular constituent. It often operates as a regulatory cofactor for enzymes and receptors. Furthermore, it possesses inherent characteristics such as high spin number, lengthy electronic relaxation time, and unstable water exchange. Mn^+2^ ions, with five unpaired electrons, could shorten the T_1_ of water protons, enhancing the T_1_ signal intensity^12–15^. In addition, Mn has a small T_2_ effect, reducing the signal intensity and producing dark images. It has critical roles in mitochondrial function and it could be accumulated in mitochondria-rich cells such as hepatocytes. Therefore, it could be applied as a proper CA for imaging of mitochondria-rich organs, including liver, pancreas, and kidney^8,16^. Mn-based CAs could be mainly divided into two categories, including Mn^2+^-bearing complexes and manganese oxide NPs. Manganese in its ionic form (Mn^+2^) has a short plasma half-life, leading to poor stability. A high level of this ion can result in the neurodegenerative abnormalities and can damage the brain. Therefore, using a Mn^+2^ complex as an imaging CA is not entirely without any issues. Different manganese oxides, at different oxidation states, including MnO, MnO_2_, Mn_3_O_4_, and MnO_x_, have been reported so far. Although MnO NPs have a good T_1_ effect in MRI, their long-term retention in the reticuloendothelial system could be toxic. Moreover, Mn_3_O_4_ NPs with a mixed valence of + 2 and + 3, exhibit no effective T_1_ relaxation, due to the fewer unpaired electrons and shorter electron spin relaxation time compared to the divalent Mn ions. While, MnO_2_ NPs could react with GSH in the tumor environment and Mn^4+^could be reduced to Mn^2+^, enhancing T_1_ MRI. Therefore, among different manganese oxides, MnO_2_ nanostructures could be considered as promising CAs for improved imaging and therapeutic applications.

Using natural enzymes as catalysts in biological systems have inherent disadvantages such as high cost, easy denaturation and inactivation, which limit their usage. To overcome these limitations, synthetic enzymes, by mimicking the function of natural enzymes, have been considered as promising alternatives. In particular, nanozymes can mimic the activity of the enzymes while maintaining the unique physical and chemical properties of nanomaterials^17^. Moreover, nanozymes can serve as CAs in tumor imaging and medical diagnosis^18^. Recently, MnO_2_ nanostructures have attracted much attention as nanozyme candidates. It has been reported that MnO_2_ has single oxidase-like or peroxidase-like activities. They have attracted attention of the researchers due to the lattice oxygen defects in their structure, plenty of natural reserves, low cost, non-toxicity, good biological safety, and biocompatibility^19,20^. Liu et al.^17^ found that MnO_2_ NPs show peroxidase- and oxidase-like activities. Under the Fenton reaction with H_2_O_2_, MnO_2_ NPs produce the most harmful reactive oxygen species (ROS), which could be used against cancerous cells^21–23^. These findings have encouraged many researchers to employ MnO_2_ NPs as safe therapeutic agents in cancer theranostics.

Tannic acid (TA) is an organic polyphenolic compound with interesting characteristics such as low cost, non-toxicity, environmentally friendly nature, and excellent metal reduction and metal chelation properties^24–26^. It was used as a porogen to prepare mesoporous silica^27^ and porous Fe_3_O_4_ NPs^25,28^. In the light of the above findings, in this work, we first aimed to find a new method to reach hierarchical mesoporous Mn-based NMs; and then we aimed to investigate the impact of morphology, surface area and porosity of two different Mn-based NMs on the MR contrast enhancement as well as nanozyme activity. The interesting features of TA as a natural template inspired us to develop a simple method for synthesizing novel hierarchical flower-like mesoporous MnO_2_ nanomaterials (MNMs). The flower-like MNMs (M1-MnO_2_) and spherical MnO_2_ (M2-MnO_2_) were synthesized and compared in term of the crystallinity, size, morphology, surface area, porosity, and magnetic properties. The nanozyme activity (peroxidase- and oxidase-like activities), cytocompatibility, and cellular uptake of the MNMs were also evaluated. Furthermore, the r_1_ relaxivity and intensity of T_1_-weighted images were investigated to study the effect of different parameters on the maximum contrast enhancement. To the best of our knowledge, this is the first work to report a simple and environmentally friendly method for the preparation of novel mesoporous MnO_2_ NMs with flower-like morphology using TA as a template. Although there are some studies on the synthesis of MnO_2_ as a contrast agent, to the best of our knowledge, no study has been reported so far in which the efficacy of flower-like mesoporous and spherical MnO_2_ nanostructures was compared in both MRI contrast enhancement and nanozyme activity. We hope our findings can open a new way to develop novel Mn-based theranostic system for future studies.

## Experimental sections

### Materials

Chemical reagents including potassium permanganate (KMnO_4,_ 97.5%), Manganese(II) nitrate tetrahydrate (Mn(NO_3_)_2_·4H_2_O, > 97%), sodium hydroxide (NaOH, 99.9%), 3,3′,5,5′-tetramethylbenzidine (TMB), and hydrogen peroxide (H_2_O_2_, 30%) were obtained from Sigma–Aldrich Company. Tannic acid (TA) and 3-(4,5-dimethylthiazolyl-2)-2, 5-diphenyltetrazolium bromide (MTT) were supplied by Merck (Germany). MCF-7 (human epithelial breast cancer) and MCF-10A (non-malignant breast epithelial) cell lines were provided by the National Cell Bank of Iran (NCBI), affiliated with the Pasteur Institute. Dulbecco’s modified Eagle medium (DMEM), RPMI 1640 medium, and phosphate-buffered saline (PBS) were purchased from Gibco BRL (Cergy Pontoise, France).

### Synthesis of the MnO_2_ NMs through two different methods

To synthesize M1-MnO_2_ NMs, 1 gr of TA, as a template, was dissolved in 10 mL of deionized (DI) water and stirred for 5 min. Then, Mn(NO_3_)_2_·4H_2_O (1.73 gr, 7.8 mmol) and KMnO_4_ (0.73 gr, 4.6 mmol) were dissolved in 10 mL of DI water, separately. In the next step, Mn(NO_3_)_2_·4H_2_O and KMnO_4_ solutions were slowly added to the TA solution, simultaneously, via two separate dropping funnels within two hours and the mixture was heated in an oil bath to 80 °C with vigorous stirring. After adding both solutions, the reaction mixture was stirred for another four hours. The precipitate was separated using centrifugation. To remove free reactants, the precipitate was washed three times with DI water, and then dried in an oven overnight at 140 ºC. The dried solid was then calcined at 500 °C for 3 h to obtain M1-MnO_2_ NMs.

For the synthesis of semispherical M2-MnO_2_ NMs, KMnO_4_ (0.7 g, 4.4 mmol) was dispersed in 10 mL of DI water and the mixture was stirred for 5 min to give a dark purple solution. Then, NaOH (0.8 gr, 20 mmol) was added to the KMnO_4_ solution, and stirring was continued for 24 h, until the solution turned brown. The obtained NMs were precipitated by centrifugation, washed three times with DI water, and dried in a vacuum desiccator at room temperature. The product was finally calcined at 500 °C for 3 h.

### Characterization of the synthesized MNMs

UV–visible absorption spectra were acquired using a UV–visible spectrometer (Thermo Scientific, USA) in the wavelength range of 200–700 nm by using a 10 mm cuvette. Fourier transform infrared (FT-IR) spectra were obtained by FT-IR Magna 550, Nicolet using the KBr plates between 400 and 4000 cm^−1^. The X-ray diffraction (XRD) patterns were recorded on a Rigaku Ultima IV diffractometer, using Cu Kα radiation (λ = 0.1542 nm), operated at 40 kV and 40 mA. The crystallite size was calculated by the Debye-Scherer equation (Eq. 1), where D is the particle size, λ is the X-ray wavelength, K is the Scherer’s constant (K = 0.94), β is full width at half maximum (FWHM), and θ is the angle of diffraction.

1 $${\text{D}} = \frac{{{\text{K}}\uplambda }}{{\upbeta \,{\text{Cos}} \,\uptheta }}$$

The X-ray photoelectron spectroscopy (XPS) was measured on an X-ray photoelectron spectrometer using Al Kα X-ray radiation source. The morphology of the MNMs was determined by field emission scanning electron microscope (FESEM, Tescan/Mira, Czech Republic). The energy dispersive X-ray spectroscopy (EDX, Shimadzu, Japan) was used to determine the elemental composition of the synthesized MNMs. The hydrodynamic diameter of MNMs was obtained from dynamic light scattering (DLS) measurements by a Zetasizer ZEN3600 (Malvern Instruments). The surface areas of the MNMs were analyzed using the Brunauer Emmett Teller (BET) method. The pore volume and pore size distribution were also determined using the Barrett–Joyner–Halenda (BJH) method. To measure the magnetic properties of the samples, a vibrating sample magnetometer (VSM, MDKB model) was used against an applied field of ± 20 kOe.

### Peroxidase- and oxidase-like activities of the MNMs

The peroxidase-like activity of the MNMs was investigated by the catalytic oxidation of TMB in the existence of H_2_O_2_. Briefly, certain amounts of M1-MnO_2_ and M2-MnO_2_ NMs were separately suspended in phosphate-citrate buffer (0.15 M) to obtain different concentrations of the samples (1, 5, 10, 25, 50, 100 µg/mL). 200 μL of the each sample was added to each well of a 96-well plate along with 2 μL of TMB solution (1.25 mM, ethanol solution) and 4 μL of H_2_O_2_. Then, the plate was vibrated in the dark to provide a homogeneous mixture, and the UV–visible spectrum (500–800 nm) of the samples was recorded using a microplate reader. For each sample, we varied one of the parameters while keeping the other parameters constant to investigate the effect of pH, temperature, and sample concentration on the catalytic oxidation reaction of TMB. Absorbance at 652 nm was measured after the reaction for 10 min using UV–visible spectroscopy. The relative activity (%) was used as the index to optimize the experimental conditions, which was calculated through dividing the current absorbance by the maximum absorbance at 652 nm related to the specific experimental conditions. Subsequently, the TMB substrate was used to evaluate the oxidase-like activity of the MNMs in the absence of H_2_O_2_. The experiment was performed using 200 μL of the samples suspended in phosphate-citrate buffer and 2 μl of TMB. After incubation for 10 min, the UV–visible absorption spectroscopy (500–800 nm) was recorded for each sample. The effect of pH, temperature, and different concentrations of the samples was also investigated as described in peroxidase-like activity.

### Cell cytotoxicity and apoptosis assay

The cytotoxicity of MNMs against MCF-7 and MCF-10A cell lines, was assessed using MTT method according to the standard protocol^29^. The cells were seeded in 96-well cell culture plates and incubated in DMEM and RPMI 1640 medium at 37 °C (cell density of 4 × 10^3^) with 5% CO_2_ for 24 h. The cells were then exposed to a sample solution of the MNMs prepared at various concentrations of 0 (untreated), 10, 25, 50, and 100 µg mL^-1^. The treated cells were then incubated for an additional 24 and 48 h. After removing the medium solution and washing the cells 3 times with PBS, 200 µL of MTT solution (5 mg mL^-1^) was added to each well and the wells were then incubated for 4 h. The MTT solution was then replaced with DMSO (200 µL/well) in the dark. The plate was placed on a shaker for 15 min. Survival of the treated cells in each well was measured using the optical density (OD) at 570 nm in wells of an ELISA plate reader (Stat Fax 2100, USA) and normalized to the untreated control cells. The assay was evaluated with three replicates (n = 3).

Flow cytometry was also performed to define the cell death mechanism for treated and untreated MCF-7 cells. MCF-7 cells were incubated in a 6-well plate (3 × 10^5^ cells per well) with the MNMs (50 μg/mL) for 24 h. Untreated MCF-7 cells were considered as the control. Collected cells were stained with fluorescein isothiocyanate (FITC)-annexin V and propidium iodide (PI) apoptosis detection kit (Hoffman-La Roch Ltd, Basel, Switzerland). After 15 min, the ratio of apoptotic cells was determined by a flow cytometer (Partec CyFlow^®^).

### Cellular uptake assay

Cell internalization of MNMs was examined with a fluorescence microscope (BD FACS Calibur, San Jose, CA, USA). Briefly, 5 mg of rhodamine was dissolved in 1 mL of DI water. 1 mL of the MNMs suspensions were mixed with rhodamine solution overnight at room temperature and dark. Then, sodium carbonate buffer (1 M) was added to the mixture and stirring was continued for 20 min. Finally, unbound rhodamine was removed with an Amicon filter (molecular weight cut-off 100 kDa, Millipore, UK). After that, rhodamine-labeled M1-MnO_2_ and M2-MnO_2_ NMs were incubated with MCF-7 and MCF-10A cell lines (5 × 10^5^ cells per well) for 4 h and their cellular uptake was evaluated.

In addition, to measure the content of M1-MnO_2_ and M2-MnO_2_ NMs in MCF-7 and MCF-10A cells, the cells were seeded in Petri dishes (Ø 6 cm) with a density of 5 × 10^5^ cells. After 48 h of seeding, the cells were incubated with MNMs (50 μg/mL) for 6 h. Then, the cells were washed three times with 10 mL of cold PBS and detached with trypsin/EDTA. The Mn content in each cell line was determined by ICP-MS. Protein concentration, proportional to the number of cells, was defined from cell lysates by the Bradford method.

### Relaxivity measurement

MRI measurements using a 1.5 T MRI scanner (Prisma, Siemens Healthcare, Erlangen, Germany) with a head neck coil at room temperature were performed to determine the potential use of the prepared MNMs as MRI contrast agents. The M1-MnO_2_ and M2-MnO_2_ NMs were dispersed on the agarose gel at various concentrations (0.04, 0.08, 0.16, 0.32, and 0.64 mM). Dotarem was used at the same concentration as the control group. The T_1_-weighted image was obtained with the following parameters using a conventional spin echo sequence: TR/TE = 50, 200, 400, 600, 800, 1100, 1300, 1500, 1800, 2000/11 ms, slice thickness = 5 mm, field of view (FOV) = 250 mm × 250 mm, field of view = 128 × 128 mm^2^, matrix size of 256 × 256. The T_2_-weighted images were obtained using a multi-spin echo sequence with the same parameters as T_1_-weighted images acquisition except TR/TE = 3000/10, 30, 60, 90, 130, 170, 210, 240, 270, 350 ms, slice thickness = 5 mm. After image acquisition, the region of interest (ROI) signal strength was defined by ImageJ software (version 1.41o). The relaxivity measurements were repeated three times for T_1_ and twice for T_2_ measurements to ensure accurate assessments. These repeated measures were averaged to obtain single T1 and T_2_ values. The T_1_ and T_2_ relaxation rate (R_1_ and R_2_) was determined using the optimal single exponential function. Finally, r_1_ and r_2_ relaxation capacity was determined by linear curve fitting of R_1_ (1/T_1_) and R_2_ (1/T_2_) versus the MNMs concentrations.

### Cellular MR imaging

MCF-7 and MCF-10A cell lines were grown in cell culture flasks under standard conditions (37 °C and 5% CO_2_), and the plates were incubated in DMEM and RPMI 1640 medium, respectively. MCF-7 and MCF-10A cells (10 × 10^6^ cells/well) were exposed to different concentrations (0, 1, and 10 μg mL^−1^) of M1-MnO_2_ and M2-MnO_2_ NMs for 6 h. Following incubation, the cells were washed three times with PBS buffer and re-suspended in PBS buffer before MR imaging. Cell phantoms were prepared using 10 × 10^6^ cells in agarose gel and placed in a rectangle tube with 0.5% agarose gel. All MRI measurements were performed with a 1.5 Tesla MRI system. For obtaining T_1_-weighted images, a spin-echo sequence was performed using the following parameters: TR/TE = 500/12 ms, matrix size = 220 × 320, slice thickness = 3 mm, FOV = 82 × 120 mm, bandwidth = 140 Hz/Px. Finally, R_1_ (1/T_1_) values were estimated by a monoexponential fitting algorithm.

### Statistical analysis

The one-way analysis of variance (ANOVA) was used to determine statistical significance using the GraphPad Prism version 9.1.1 (GraphPad, San Diego, CA). All the data were reported as mean ± standard deviation (SD). *P* value < 0.05 was considered statistically significant.

## Results and discussion

### Characterization of the synthesized MNMs

**Figure 1:**
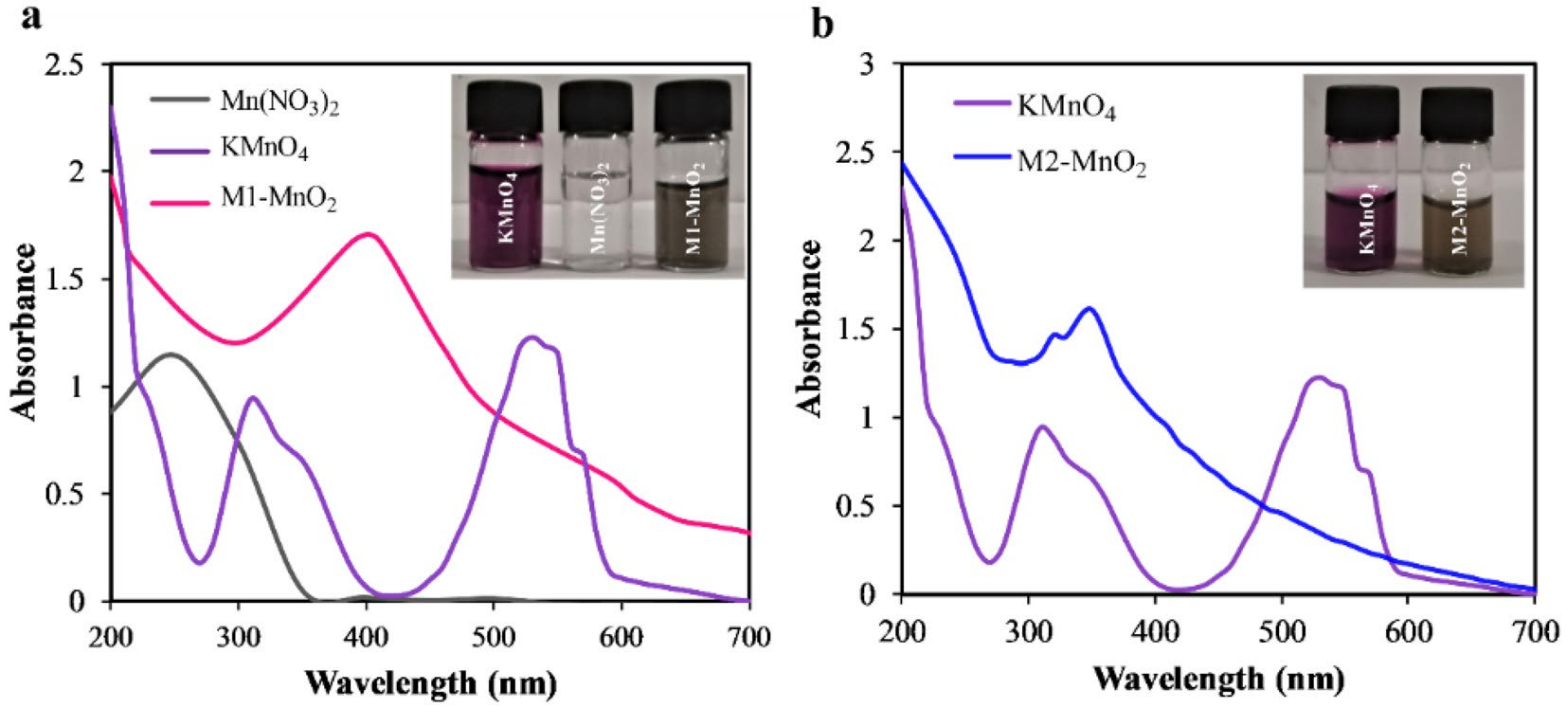
UV–visible spectra of (**a**) M1-MnO_2_ and (**b**) M2-MnO_2_ NMs.

UV–visible analysis was employed to evaluate completion of the reaction. After completion of the reaction, the characteristic absorption band of KMnO_4_ was disappeared at about 530 nm (Fig. 1a). In the meantime, the purple color of the KMnO_4_ solution was disappeared and, the color of the mixture turned dark brown. Also, the characteristic absorption band of Mn(NO_3_)_2_ in the range of 200–300 nm disappeared. The synthesized M1-MnO_2_ NMs showed a maximal absorbance around 410 nm, ascribed to the d–d transition of Mn ions in MnO_2_^30,31^. This is a clear indication of the formation of MnO_2_ NMs. Figure 1b shows that after adding NaOH to the KMnO_4_ solution, the characteristic absorption band of KMnO_4_ at 530 nm was also disappeared, and the color of the mixture turned brown. The synthesized M2-MnO_2_ NMs showed maximum absorption at about 360 nm. The significant blue shift for M2-MnO_2_ compared to M1-MnO_2_ could be related to the different morphology of these nanostructures.

**Figure 2:**
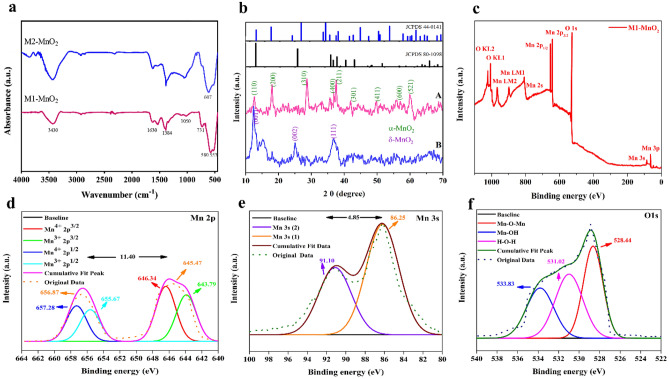
(**a**) FT-IR spectra of M1-MnO_2_ and M2-MnO_2_ NMs. (**b**) XRD patterns of (**A**) M1-MnO_2_, (**B**) M2-MnO_2_. (**c**) A wide-scan XPS spectrum of M1-MnO_2_. Core level high-resolution XPS spectrum of (**d**) Mn 2*p*, (**e**) Mn 3*s*, and (**f**) O 1*s* energy levels.

The chemical composition of the prepared MNMs was investigated by FTIR analysis. A broad band was appeared at around 3430 cm^−1^ in both spectra, which could be attributed to the O–H stretching vibration. The weak band around 1630 cm^−1^ could be also related to the O–H bending vibrations (Fig. 2a)^32^. These bands could be related to the adsorbed water molecules and the free hydroxyl groups in the structure of MnO_2_ NMs. The peaks around 1384 and 731 cm^−1^ could be attributed to the vibrations of the O–Mn–O bonds in the MnO_2_ structure^33^. For both samples, the characteristic bands at 500–800 cm^−1^ were observed. The peaks that appeared at 533 and 580 cm^−1^ in the spectrum of M1-MnO_2_ could be related to the Mn–O lattice vibration^33,34^. However, the position of the characteristic bands displayed some apparent changes in M2-MnO_2_. The absorption band related to the Mn–O–Mn vibration was appeared at around 553 cm^−1^ in M1-MnO_2_ and 607 cm^−1^ in M2-MnO_2_.

XRD analysis was used to study the crystallinity of the prepared MNMs and to identify the phase of the crystals. It is well known that MnO_2_ has several polymorphs (types of α, β, γ, δ, etc.), which are different in crystal structures^35^. The XRD patterns of the samples are shown in Fig. 2b. The XRD pattern of M1-MnO_2_ NMs shows predominant peaks at 2θ = 12.59, 18.06, 28.67, 36.10, 37.54, 42.0, 49.80, 58.86, and 60.08 º, which are attributed to the (110), (200), (310), (400), (211), (301), (411), (600), and (521) crystal planes, respectively (Fig. 2bA). The presence of these diffraction peaks is ascribed to the α-MnO_2_ crystal phase, which is consistent with the database in the Joint Committee on Powder Diffraction Standards (JCPDS) card number of 44-0141^36^. No other peaks related to the impurity phases were observed. The crystallite diameter of M1-MnO_2_ calculated by Debye-Scherer was 16.94 nm. Furthermore, the XRD peaks of M2-MnO_2_ were appeared at 2θ = 12.42, 24.92, and 36.69°, which could be attributed to the crystal planes of (001), (002), and (111), respectively (Fig. 2bB). The presence of these sharp peaks in the XRD pattern of M2-MnO_2_ NMs confirmed formation of the predominant phase of δ- MnO_2_ according to JCPDS card number of 80–1098^37,38^. The crystallite diameter of M2-MnO_2_ calculated by Debye-Scherer was 14.55 nm. The results revealed that the applied method for the synthesis has a significant effect on the crystalline lattice of the prepared MnO_2_ nanostructures^37^. Moreover, the sharp diffraction peaks indicate that the MnO_2_ nanostructures are crystalline in nature.

To investigate the state of Mn, XPS analysis was performed. Figure 2c illustrates the scan XPS survey spectrum recorded from the M1-MnO_2_ NMs in the range of 0–1100 eV. The appearance of Mn binding energy peaks (2*s*, 2*p*^3/2^, 2*p*^1/2^, 3*p*, 3*s*) and O1s suggests the formation of MnO_2_ without any contaminants and impurity peaks. The high-resolution XPS spectrum of Mn 2*p* reveals the spin–orbit doublet components located at ⁓ 656.87 and 645.47 eV, corresponding to the different binding energies of Mn 2*p*^1/2^ and Mn 2*p*^3/2^, respectively (Fig. 2d). The spin energy separation between these two peaks is 11.40 eV, which is consistent with the literature reported XPS spectrum of MnO_2_^39,40^. Moreover, the broad Mn 2*p*^1/2^ and 2*p*^3/2^ peaks are deconvoluted into two peaks due to the overlapping of Mn^3+^ and Mn^4+^ ions. Additionally, the Mn 3*s* spectrum consists of two peaks positioned at ∼ 91.10 and 86.25 eV, corresponding to Mn 3*s* (2) and Mn 3s (1), respectively, with an energy separation of 4.85 eV (Fig. 2e). The splitting of Mn 3*s* spectrum can be due to the coupling of 3*s* electron and 3*d* valence-band electrons. Therefore, the results reveal that the sample includes mainly Mn^4+^ with some Mn^3+^ ions. In addition, the three binding energy peaks of the O 1s spectrum at 528.24, 531.02, and 533.83 eV, attributed to the Mn–O–Mn, Mn–OH, and H–O–H bonds, respectively (Fig. 2f), indicate the − 2 oxidation state of oxygen^13^.

**Figure 3:**
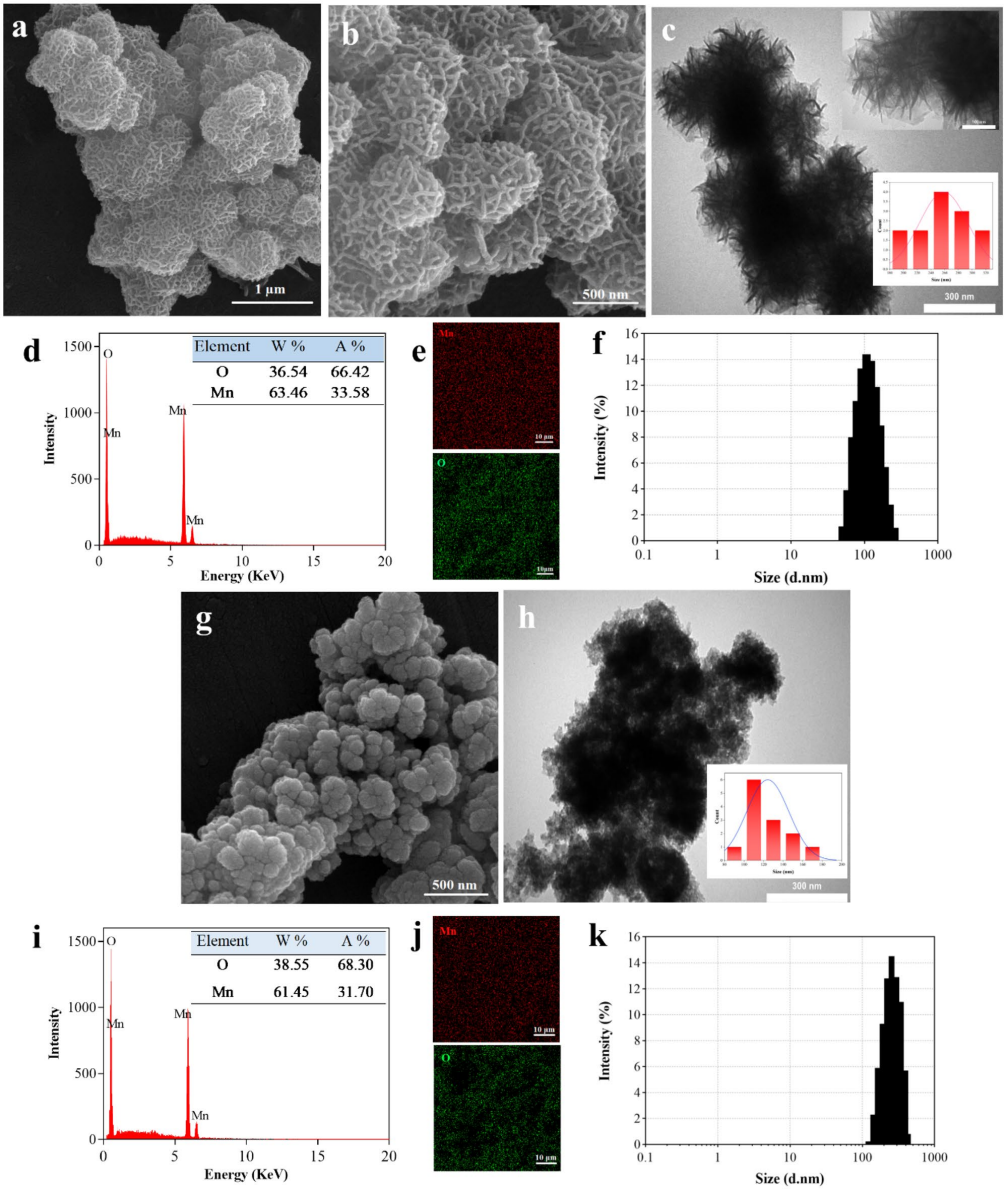
FESEM images of (**a, b**) M1-MnO_2_ (scale bar: 1 µm and 500 nm) and (**g**) M2-MnO_2_ (scale bar: 500 nm). TEM images of (**c**) M1-MnO_2_ and (**h**) M2-MnO_2_ (scale bar: 300 nm, inset in **c**: 100 nm). EDX elemental analysis of (**d**) M1-MnO_2_ and (**i**) M2-MnO_2_; Insets: Quantitative analysis. Elemental mapping of (**e**) M1-MnO_2_ and (**j**) M2-MnO_2_ (scale bar: 10 μm): red and green points represent manganese and oxygen, respectively. Hydrodynamic diameter of (**f**) M1-MnO_2_ and (**k**) M2-MnO_2_ characterized by DLS.

FESEM was used to evaluate morphology of the samples. As shown in Fig. 3a,b, M1-MnO_2_ NMs showed a porous flower-like morphology, which created a porous structure with interconnected pores. The possible mechanism for the formation of this morphology could be the growth of manganese oxide nuclei during the Ostwald ripening process, resulting in the formation of a flower-like structure^41^. M2-MnO_2_ NMs showed a near-spherical morphology, which tended to agglomerate (Fig. 3g). The calcination process increases the crystallinity. Also, it removes impurities resulting in the crystalline materials with better properties compared to their amorphous structures^42^. Moreover, an organic template such as TA, which is removed in the calcination process, can be used to prepare nanostructures with a unique porous morphology^24^. The corresponding TEM images of the MNMs are shown in Fig. 3c and Fig. 3h. Through the analysis of TEM images by imageJ software, the average size of flower-like and near-spherical MNMs was estimated to be 259 and 115 nm, respectively. The elemental composition of the MNMs was identified using EDX analysis. As shown in Fig. 3d, neither K nor N signals were detected in the EDX spectrum of M1-MnO_2_, and only Mn and O atoms were present, confirming formation of M1-MnO_2_ NMs without any impurity. The absence of C peaks in the EDX spectrum confirmed the removal of TA during the calcination process. Similarly, the EDX spectrum of M2-MnO_2_ showed no peaks related to the presence of K (Fig. 3i). The data extracted from the EDX analysis of the MNMs (tables inset in Fig. 3d,i) indicates that the prepared MNMs have a composition of Mn and O atoms, which corresponds to the MnO_2_ formula^43^. The elemental mapping images showed uniform distribution of Mn as well as O in the prepared MNMs (Fig. 3e,j). Besides, the hydrodynamic diameter of the MNMs in water was determined by DLS analysis (Fig. 3f,k). The mean size diameter of M1-MnO_2_ and M2-MnO_2_ NMs were 285 ± 24.17 and 129 ± 10.48 nm, respectively. Average size of the NPs used in MRI is about 3.0 to 350 nm^44^. Since the colloidal stability of nanostructures is a critical issue for biological application, the stability of flower-like MnO_2_ NMs was also evaluated by DLS analysis in different media (Fig. S1). The size of flower-like MnO_2_ NM did not change significantly during 7 days of incubation in both PBS (pH = 7.4) and DMEM containing 10% FBS. However, flocculation of flower-like MnO_2_ in DI water resulted a little change in the hydrodynamic diameter, indicating the relatively poor stability of flower-like MnO_2_ in water over time. The results confirmed the stability of flower-like MnO_2_ for in vivo biomedical applications.

**Figure 4:**
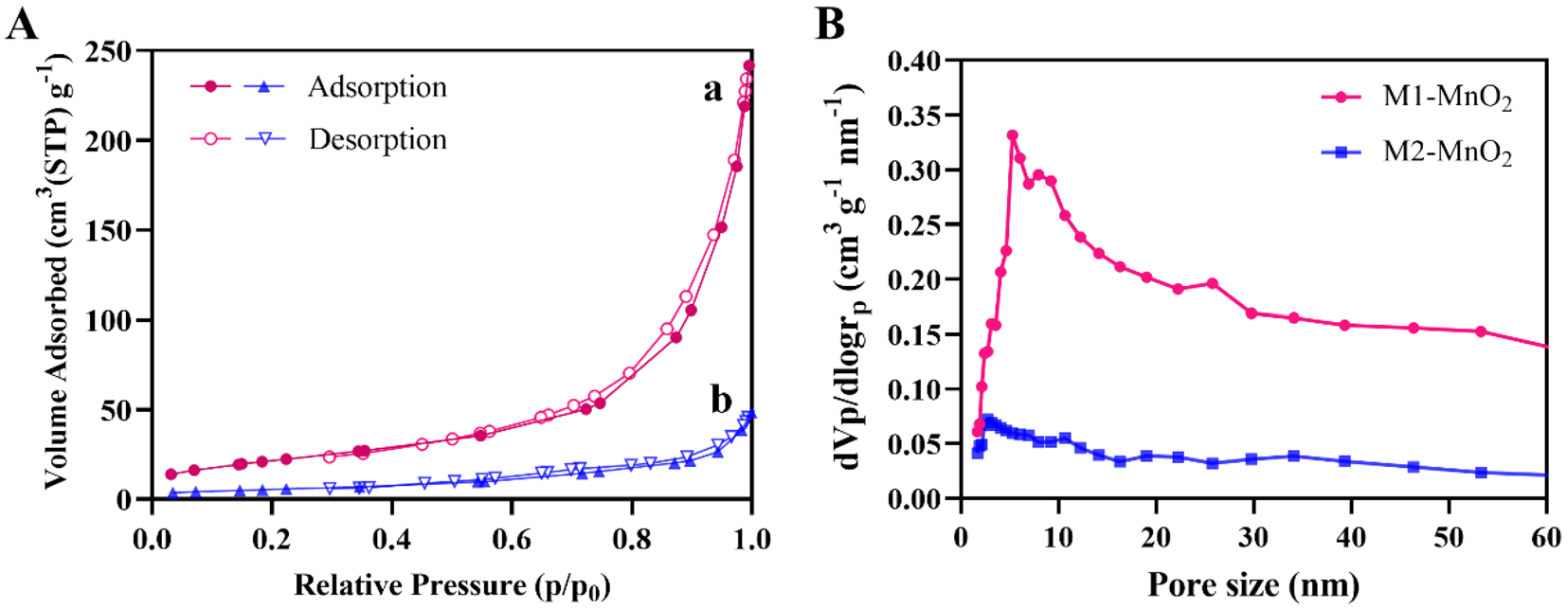
(**A**) The N_2_ adsorption–desorption isotherms of (**a**) M1-MnO_2_ and (**b**) M2-MnO_2_ in different morphologies. (**B**) BJH pore size distribution.

The surface area and pore size distribution of the synthesized MNMs with different morphologies are shown in Fig. 4. The BET surface area was investigated by measuring the N_2_ adsorption–desorption isotherms, and the pore size distribution was determined from the BJH analysis of N_2_ adsorption isotherms^45^. The measurements showed that the BET surface area of M1-MnO_2_ NMs was 78.47 m^2^ g^−1^ and the pore size distribution was 5.27 nm. The BET surface area of M2-MnO_2_ NMs was estimated to be 20.05 m^2^ g^−1^ and there was a small pore distribution peak at 2.73 nm. Values of the surface area, total pore volume, and mean pore size of the MnO_2_ samples can be found in Table 1. The results suggest that with the increase of the specific surface area, the pore volume and pore size increase. This could be due to the presence of interconnected pores^46^. M1-MnO_2_ NMs showed the highest BET surface area, probably because the pores were large enough to host N_2_ molecules^45^. The morphology of M2-MnO_2_ which consists of agglomerates of near-spherical particles can negatively affect the formation of pores in the material and reduce the total pore volume, which leads to N_2_ molecules to be adsorbed mainly on the external surface. It could also be implied that after calcination and removal of TA, the surface area increases due to the appearance of new pores, which increases total pore volume and average pore size^47^. Furthermore, porous nanostructures possess significant properties, such as high surface area, making them valuable for various applications^48^.

**Table 1:** Surface areas and pore structures of the synthesized MNMs with different morphologies.

Samples	BET surface area (m^2^ g^−1^)	Total pore volume (cm^3^ g^−1^)	BJH pore size (nm)
M1-MnO_2_	78.47	0.35	5.27
M2-MnO_2_	20.05	0.07	2.73

The magnetic hysteresis (M-H) curve of the MNMs at 300 K using VSM method is shown in Fig. S2. The MnO_2_ nanostructures, synthesized in this work, displayed an early linear relationship between their magnetization and applied field, indicating that the MNMs had the paramagnetic state^49^. However, a small coercivity at the low applied manetic fields (− 2 kOe − 2 KOe) was detected in the M2-MnO_2_ sample, suggesting a very weak ferromagnetic-like state^50^. The magnetic saturation (Ms) of M1-MnO_2_ and M2-MnO_2_ was 0.2 and 0.4 emu/g, respectively. Based on previous studies, it is believed that the Ms of magnetic NMs depends not only on the particle size but also on their surface properties^51,52^. Flower-like NMs behave differently compared to similar single-core nanoparticles. They are composed of different cores having similar aspects, so they show better magnetic properties due to the reduced anisotropy and large surface area^53,54^. Therefore, the better magnetic performance of flower-like M1-MnO_2_ NMs could be attributed to their large specific surface area compared to M2-MnO_2_ NMs.

### Dual-enzyme mimetic activities of MNMs

**Figure 5:**
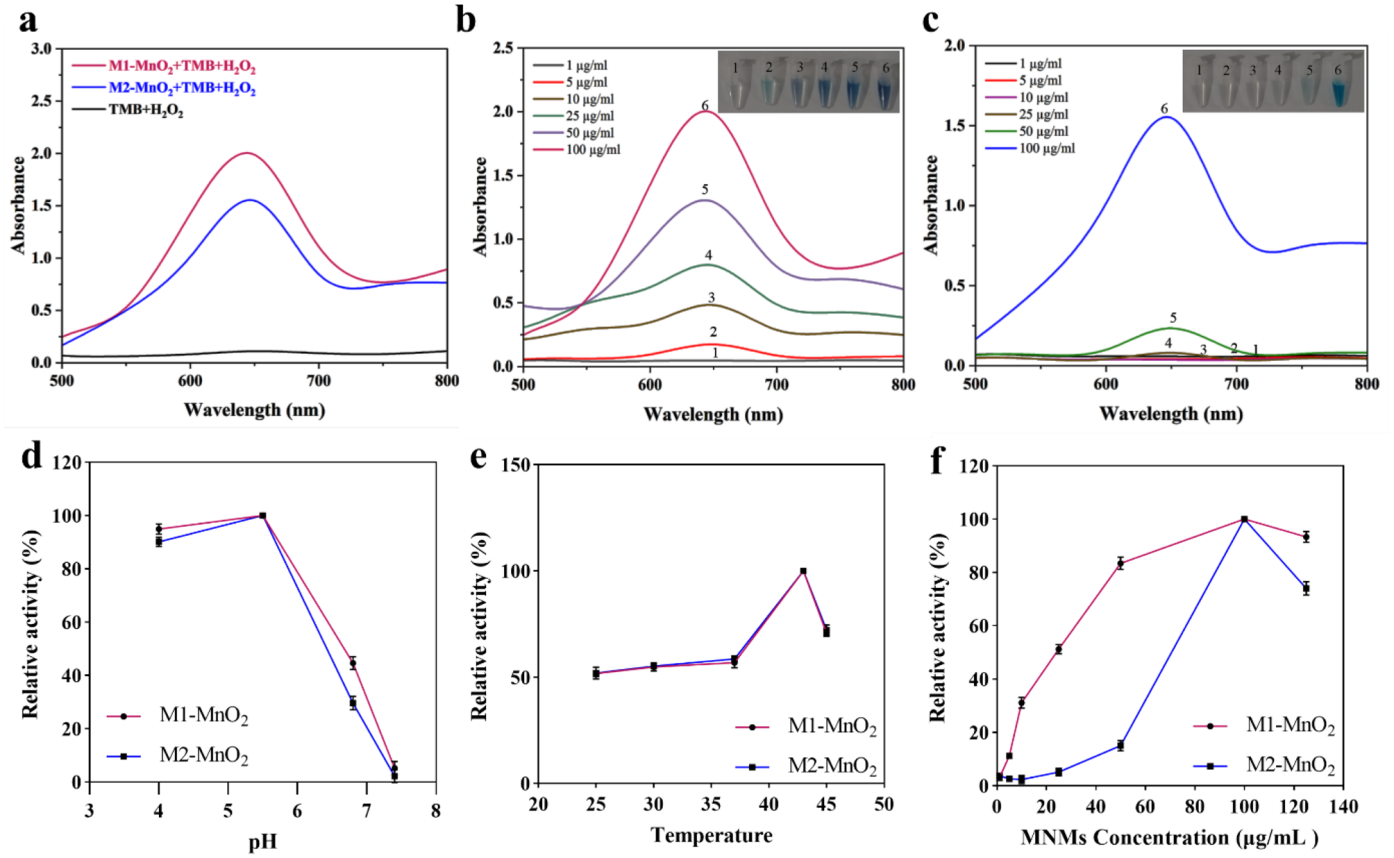
(**a**) Comparison of peroxidase-like activity between M1-MnO_2_ and M2-MnO_2_ after adding 1.25 mM TMB in the presence of H_2_O_2_. The absorbance spectra of (**b**) M1-MnO_2_ and (**c**) M2-MnO_2_, where the peak at 650 nm indicates the oxTMB (Inset: visual color changes of peroxidase-like activity proportional to increasing the concentration). Effects of (**d**) pH, (**e**) temperature, and (**f**) concentration of the MNMs. The maximum activity in each graph of the same variable was set to 100% (n = 3, *P* < 0.05).

Peroxidase- and oxidase-like nanozymes catalyze the oxidation of TMB to an aqueous oxidized product (oxTMB) in the presence and absence of H_2_O_2_, respectively, with a major absorption peak at 652 nm. M1-MnO_2_ NMs with flower-like morphology showed relatively higher peroxidase-like activity than M2-MnO_2_ NMs with near-spherical morphology (Fig. 5a). The properties of the nanoenzymes depend on the reaction conditions^55^. As shown in Fig. 5b,c, the absorbance at 652 nm increased with increasing the concentration of the MNMs. The effect of pH, temperature, and concentration of the samples on peroxidase-like activity were also evaluated, and absorbance at 652 nm was recorded for each condition. As shown in Fig. 5d, the peroxidase-like activity reached a higher level in an acidic buffer (pH 4–5.5) than near neutral buffer. The highest peroxidase-like activity was obtained at 43 °C (Fig. 5e). The optimal dose of M1-MnO_2_ and M2-MnO_2_ NMs was 100 μg/mL (Fig. 5f). Therefore, the optimal conditions for the maximum peroxidase-like catalytic activity were observed as pH = 5.5, T = 43 °C, and 100 µg/mL of the MNMs.

**Figure 6:**
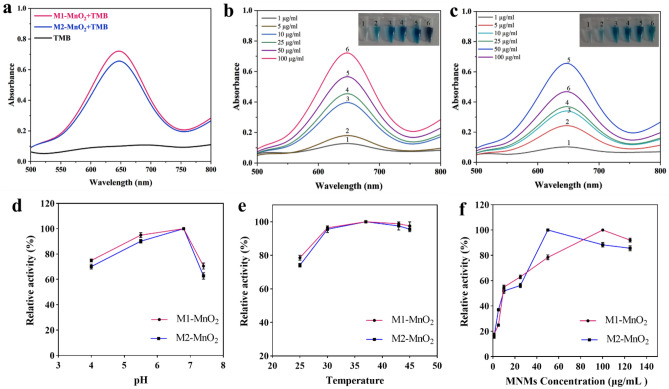
(**a**) Comparison of oxidase-like activity between M1-MnO_2_ and M2-MnO_2_ after adding TMB (1.25 mM) in the absence of H_2_O_2_. The absorbance spectra of (**b**) M1-MnO_2_ and (**c**) M2-MnO_2_, where the peak at 650 nm indicates the oxTMB (Inset: visual color changes of oxidase-like activity proportional to increasing the concentration). Effects of (**d**) pH, (**e**) temperature, and (**f**) concentration of the MNMs. The maximum activity in each graph of the same variable was set to 100%. (n = 3, *P* < 0.05).

Subsequently, TMB was used as a substrate to investigate the oxidase-like activity of the MNMs in the absence of H_2_O_2_. Figure 6a shows that both M1-MnO_2_ and M2-MnO_2_ NMs catalyze the rapid oxidation of TMB in the absence of H_2_O_2_. The intensity of blue color and absorption at 652 nm increased with increasing the concentration of the MNMs (Fig. 6b,c). The oxidase mimics exhibited high activity at pH = 6.8 and T = 37 °C (Fig. 6d,e). When the concentration of M1-MnO_2_ NMs was in the range of 1–100 µg/mL, the absorption increased by increasing the concentration (Fig. 6f). When the M1-MnO_2_ NMs concentration was higher than 100 µg/mL, the absorption decreased at 652 nm. This could be caused by the catalytic oxidation of oxTMB to biphenyl quinone in the presence of excess amount of M1-MnO_2_^56^. In the case of M2-MnO_2_ NMs, the maximum oxidase-like catalytic activity was at a concentration of 50 μg/mL and the absorption decreased above this concentration. This could be due to the high tendency of near-spherical M2-MnO_2_ NMs to agglomeration, affecting the catalytic activity of nanozymes^55^. Accordingly, the optimal conditions for the maximum oxidase-like catalytic activity were pH = 6.8, T = 37 °C, 100 µg/mL for M1-MnO_2_ and 50 µg/mL for M2-MnO_2_ NMs. The results suggest that M1-MnO_2_ NMs show more peroxidase- and oxidase-like activities compare to M2-MnO_2_ NMs. These characteristics are mainly due to its morphology with a large BET surface area, allowing it to have a higher affinity towards TMB^57^. The crystal structure, morphology, and specific surface area significantly affect the activity of enzyme mimics^56,58^. It has been found that a larger surface area and better dispersion of NMs significantly improve their catalytic activity^55^. Altogether, flower-like M1-MnO_2_ NMs can maintain aceptable nanozyme activity in a range of pH, temperature, and concentration, providing a bright prospect for future applications, especially cancer therapy.

### Cell cytotoxicity and apoptosis assay

**Figure 7:**
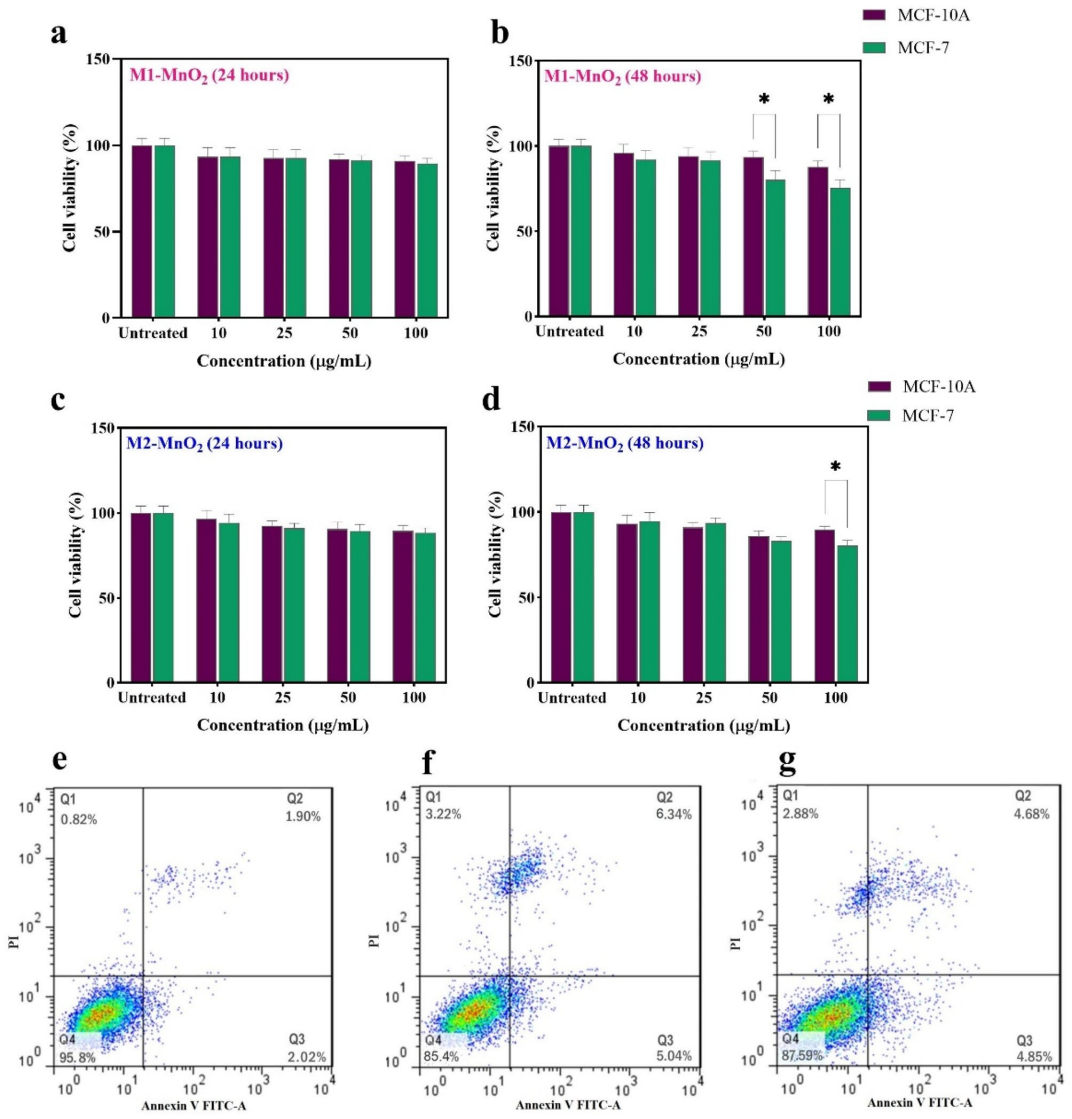
Cell viability of MCF-10 A and MCF-7 cells after incubation with M1-MnO_2_ NMs (**a**: 24 and **b**: 48 h) and M2-MnO_2_ NMs (**c**: 24 and **d**: 48 h) at varying concentrations (*P < 0.05). Flow cytometry assay shows the apoptotic rates of MCF-7 cells after treatment; (**e**) control, (**f**) M1-MnO_2_, and (**g**) M2-MnO_2_ NMs; Q1: necrotic cells, Q2: late-stage apoptotic cells, Q3: early-stage apoptotic cells, Q4: viable cells.

To evaluate safety of the MNMs, a cytotoxicity assay was performed using MTT in both cancerous MCF-7 and normal MCF-10A cell lines. The dose-(10, 25, 50, and 100 µg/mL) and time-dependent (24 and 48 h) cytotoxicity profiles were obtained for each sample, M1-MnO_2_ and M2-MnO_2_. As shown in Fig. 7, no significant cytotoxicity was observed at various concentrations of M1-MnO_2_ and M2-MnO_2_ during 24 h of incubation (Fig. 7a,c). Even at a concentration of 100 µg/mL, the cell viability remained above 88%, indicating the biocompatibility of the MNMs. In our study, the decrease in cell survival with increasing the concentration was not significant in both samples compared to the control group (untreated cells), making the MnO_2_ NMs more favorable for biomedical applications compared to Mn^2+^-based CAs. Besides, by increasing the incubation time to 48 h, the cell viability decreased with a concentration-dependent trend. Cell viability in normal cells after 48 h of incubation for both samples was higher than that of in cancer cells. The highest cytotoxicity of M1-MnO_2_ and M2-MnO_2_ NMs on cancer cells was observed at concentrations above 50 and 100 μg/mL, respectively (*P < 0.05) (Fig. 7b,d). At a concentration of 100 μg/mL, a decrease in cell viability in cancer cells treated with M1-MnO_2_ was probably due to the acidic internal environment of cancer cells. In addition, we demonstrated that M1-MnO_2_ NMs had dual enzyme-mimetic activities at acidic pH (5.5–6.8). The cell viability of MCF-7 cells in the presence of spherical MnO_2_ was reported to be 45%^59^. Wang et al.^60^ demonstrated that the nanoflowers had little cytotoxicity on normal cells, but they could induce cancer cell death, indicating that the flower-like nanostructures can inhibit cancer cell growth. The performance of the flower-like mesoporous MnO_2_ NMs was better than the reported values^61,62^. Therefore, the M1-MnO_2_ NMs have the potential to be used as MRI CA to identify cancer cells, and since they are selectively cytotoxic to cancer cells, they can also be used against cancerous cells, without harming normal cells. Flow cytometry was performed to evaluate the cell death mechanism for MCF-7 cells treated with 50 μg/mL of MNMs (Fig. 7e–g). Although more than 80% of the cells survived, the late-stage apoptosis rate of the cells after incubation with flower-like M1-MnO_2_ was slightly higher than that of incubation with near-spherical M2-MnO_2_ NMs. The results clearly showed that the flower shape promotes the cytotoxicity of MNM in cancer cells. It has been reported that flower-like nanostructures can disrupt the cell wall better than spherical ones, thereby increasing the death of human endothelial cells^63–65^. Sultana et al. found that flower-shaped NPs have a higher surface area than spherical NPs, so they are more potent to interact with the cell membrane due to the spiky surface. The morphology-dependent cell death mechanism supports the hypothesis that surface roughness is responsible for cell membrane disruption, and is a critical parameter in cellular internalization of nanostructures^64^. In this study, the flower-like mesoporous M1-MnO_2_ exhibited a relatively high surface area for biological interactions, which can increase the ability to kill cancer cells. These findings are consistent with the results obtained from the MTT assay. Therefore, MnO_2_ NMs with flower shape can be a potential candidate, improving diagnostic sensitivity and therapeutic efficiency.

### Cellular uptake

**Figure 8:**
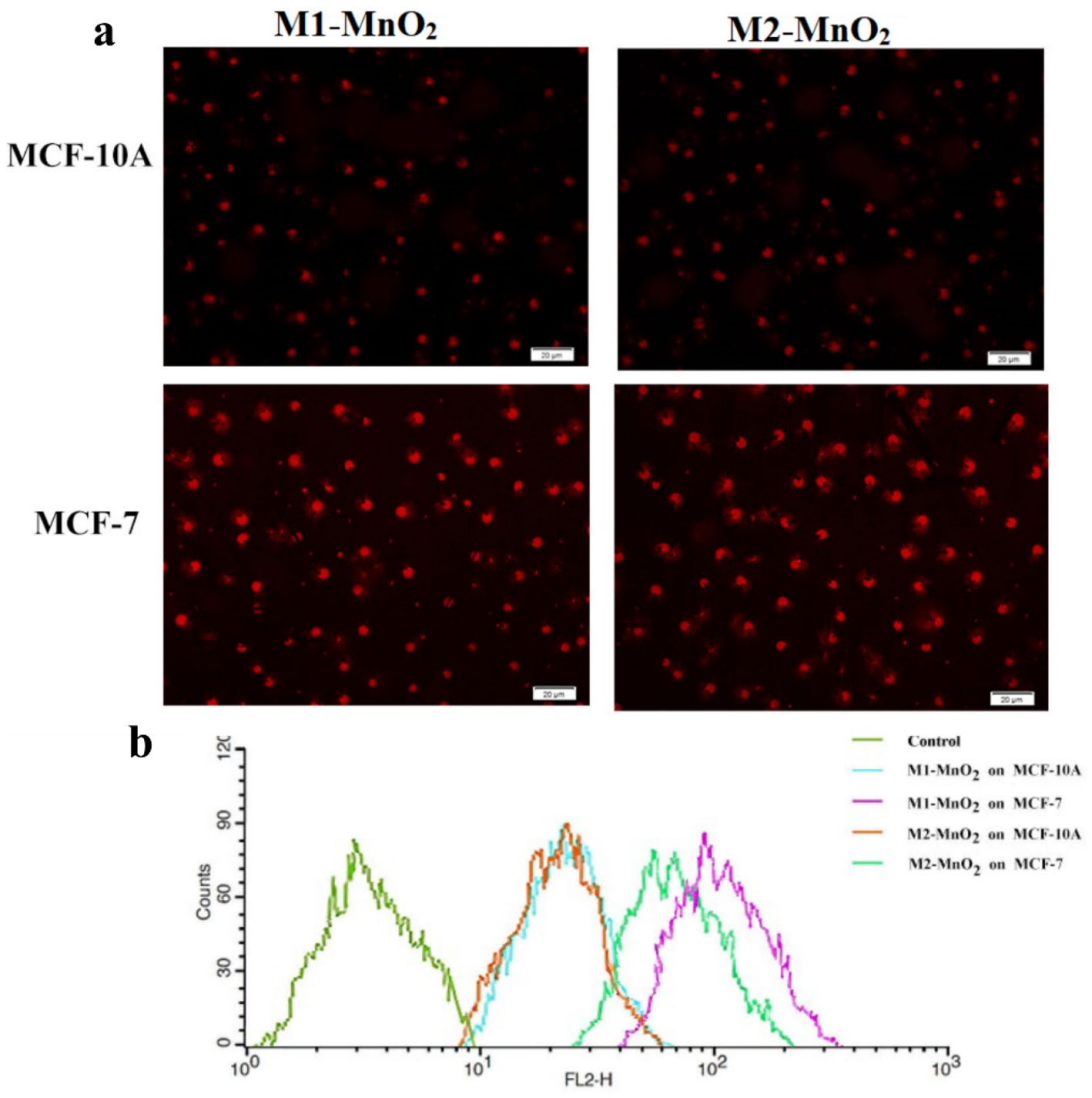
(**a**) Qualitative and (**b**) quantitative cellular uptake assays of M1-MnO_2_ and M2-MnO_2_ NMs in MCF-7 and MCF-10A cells.

Prior studies approved that the physical parameters of NPs, including size, shape, porosity, surface area, pore volume, and stable aqueous dispersibility affect the interaction between NPs and cell membrane, controlling the cell penetration^66,67^. Nanostructures with a well-defined shape are internalized by non-specific cellular uptake^68^. In this study, in vitro cellular uptake was evaluated using fluorescence microscopy and amount of M1-MnO_2_ and M2-MnO_2_ NMs taken up by MCF-7 and MCF-10A cells was estimated (Fig. 8a,b). The fluorescence intensity of cell uptake in cancer cells was stronger than normal cells. The increase in cellular uptake may be due to the higher activity of cancer cells than normal cells which could absorb the MNMs via interactions on the cell membrane. Moreover, the permeability of the nanostructures is influenced by the contact surface with the lipid layer^69^. In non-spherical structures, due to the greater contact surface, the interaction with the membrane increases, and subsequently the cell internalization efficiency improves compared to spherical structures^70^. Therefore, the higher cellular uptake of flower-like M1-MnO_2_ NMs could be due to the higher surface area and more surface roughness compared to near-spherical M2-MnO_2_ NMs. The content of M1-MnO_2_ and M2-MnO_2_ in MCF-7 and MCF-10A cells was also determined by ICP-MS. Fig. S3 shows the amount of the internalized Mn normalized to the total protein content of the cell. Internalized Mn values were significantly higher for MCF-7 incubated in the presence of M1-MnO_2_ than M2-MnO_2_ (*P* < 0.05). On the contrary, Mn content in MCF-10A cells was low, with no significant difference for two types of MNMs (*P* > 0.05). Our findings suggest that flower-like mesoporous MnO_2_ NMs exhibit more Mn content in cancer cells due to the more non-specific cellular uptake and faster internalization rate compared to the spherical shape.

### Relaxivity

**Figure 9:**
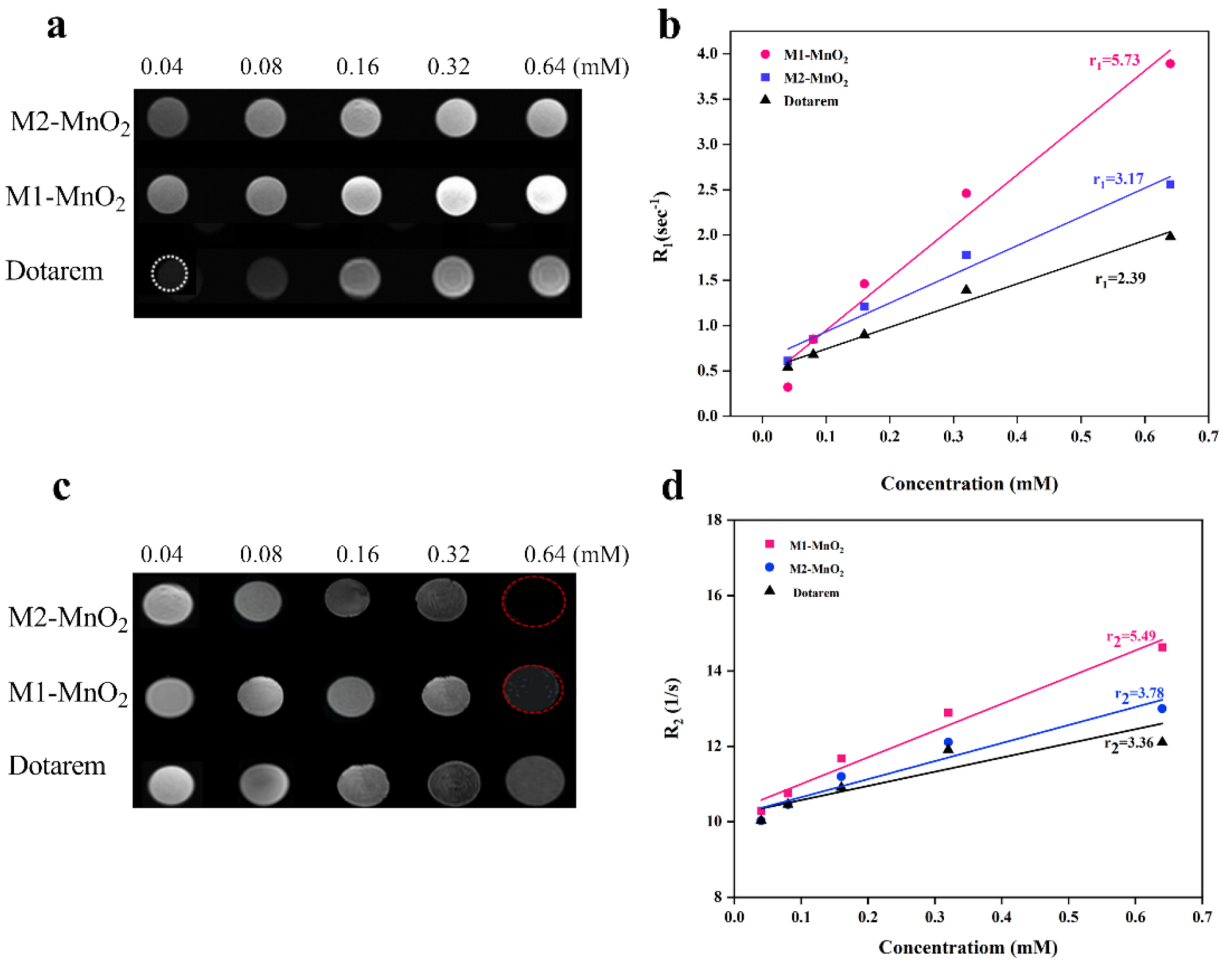
(**a**) T_1_-weighted images, (**b**) the longitudinal relaxivity, (**c**) T_2_-weighted images, (**d**) and the transverse relaxivity of M1-MnO_2_ and M2-MnO_2_ NMs dispersed in water.

To investigate the effectiveness of the MNMs as a T_1_ MRI contrast agent, T_1_-weighted images were obtained using a clinical 1.5 T MRI scanner. The M1-MnO_2_ and M2-MnO_2_ showed slightly brighter T_1_-weighted images than commercially approved MRI contrast agent (Dotarem) in a phantom model (Fig. 9a). It was found that M1-MnO_2_ and M2-MnO_2_ NMs effectively shortened the longitudinal relaxation time (T_1_) and significantly increased the signal intensity in T_1_-weighted images in comparison with Dotarem. Also, the degree of the bright contrast enhancement in T_1_-weighted images was directly related to the concentration of Mn ions. The longitudinal relaxivity values (r_1_) for M1-MnO_2_, M2-MnO_2_, and Dotarem were achieved at 5.73, 3.17, and 2.39 mM^−1^ s^−1^, respectively (Fig. 9b). The obtained r_1_ value was comparable to the previously reported Mn nanoparticles^71,72^. The difference between the prepared MnO_2_ NMs in this study could be due to the significant difference in morphologies, pore volume, specific surface area, and subsequently potential H_2_O absorption sites of the MNMs. Some studies showed that samples with large pore volume provide high r_1_^12,73^. This trend could be described by different rotational dynamics, where the large NPs are likely to rotate more slowly than smaller NPs with lower moments of inertia^74,75^. According to SEM and BET results, M1-MnO_2_ NMs have a uniform porous structure with a higher specific surface area and pore volume than M2-MnO_2_ NMs. Therefore, the morphology of M1-MnO_2_ NMs could result in the absorption of more water molecules in the structure, which leads to an increase in the r_1_ relaxivity value. The decrease in the r_1_ relaxivity value in M2-MnO_2_ compared to M1-MnO_2_ could be due to its slightly higher polydispersity. In addition, the aggregation of M2-MnO_2_ NMs could reduce the T_1_ contrast ability^76^. However, no significant signal intensity changes were observed in T_2_- weighted images of the MNMs, indicating that MNMs had a small T_2_ effect (Fig. 9c). The transverse relaxivity values (r_2_) for M1-MnO_2_, M2-MnO_2_ NMs and Dotarem were 5.49, 3.78, and 3.36 mM^−1^ s^−1^, respectively (Fig. 9d). The low r_2_/r_1_ ratio value for M1-MnO_2_ indicated that the T_1_-shortening effect was dominant over the T_2_ effect, indicating a strong contrast enhancement in the T_1_-weighted images. In this regard, the obtained results suggest that M1-MnO_2_ NMs have the potential to be used as T_1_ MRI contrast agents.

### Cellular MR imaging

**Figure 10:**
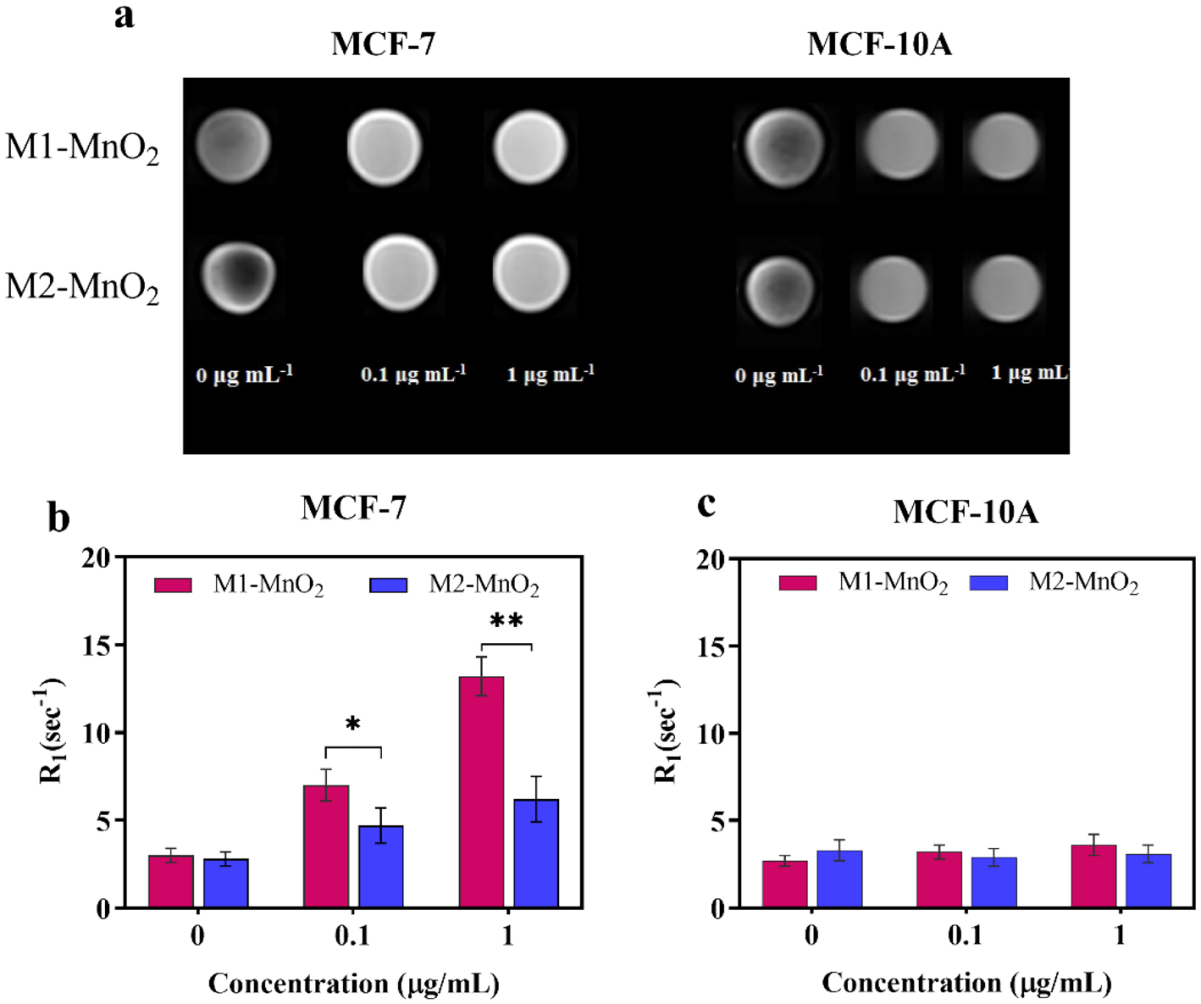
(**a**) T_1_-weighted images and longitudinal relaxation rate of (**b**) MCF-7 and (**c**) MCF-10A cells treated with M1-MnO_2_ and M2-MnO_2_ NMs at different concentrations (n = 3, *P < 0.05, **P < 0.01).

To confirm the diagnostic ability of the MnO_2_ NMs as a T_1_ MRI contrast agent, MCF-7 and MCF-10A cells were incubated with MNMs at different concentrations (0, 0.1, and 1 μg/mL), and MR signal intensity was evaluated. As shown in Fig. 10a, T_1_-weighted MR images gradually became brighter as the concentration of the MNMs increased in the treated cells. The concentration-dependent brightening effect in T_1_-weighted MR images is more obvious in the lower pH environment of MCF-7 cells, while the MR signals appear relatively weaker in MCF-10A normal cells with the natural pH of 7.4. Moreover, the longitudinal relaxation rate (R_1_) of MCF-7 cells treated with M1-MnO_2_ and M2-MnO_2_ was significantly higher than that of MCF-10A cells (Fig. 10b). The increase in R_1_ value in MCF-7 cells could be related to the accumulation of paramagnetic Mn^+2^ in MCF-7 cells, induced by the MnO_2_ nanostructures, resulted from the acidic internal environment of the cancer cells compared to the normal cells. MnO_2_ is known to be stable at neutral and basic pH, but it could be decomposed to Mn^+2^ and O_2_ under acidic pH^59^. Furthermore, since Mn^+2^ with five unpaired electrons could shorten the T_1_ of water protons and enhance the T_1_ signal intensity, the presence of Mn^+2^ in the developed MnO_2_ NMs enables T_1_-based metabolic imaging^59,77^. Figure 10 shows that the R_1_ of M1-MnO_2_ in the presence of MCF-7 cancer cells was higher than that of M2-MnO_2_ NMs, which could be ascribed to the high accessibility of water molecules to the Mn^+2^ paramagnetic centers. Hence, M1-MnO_2_ are more suitable as a T_1_ MRI contrast agent. M1-MnO_2_ may act as a promising pH-sensitive cellular and metabolic MR imaging agent, particularly useful for tumor imaging having the acidic tumor microenvironment. The obtained results of this study was comparable to what achieved in previous works (Table S1).

## Conclusion

In thgis work a simple and green method using TA as a template was introduced for the preparation of novel flower-like mesoporous MnO_2_ nanomaterials (M1-MnO_2_), with hydrodynamic size of ~ 285 nm, and relatively large surface area, high pore volume, as well as appropriate paramagnetic behavior compared to near-spherical M2-MnO_2_. The paramagnetic property of the MNMs was strongly dependent on the shape and surface area of the prepared samples, so the flower-like M1-MnO_2_ with the higher surface area showed better paramagnetic properties than near-spherical M2-MnO_2_. It was found that M1-MnO_2_ NMs had inetresting peroxidase- and oxidase-like activities. Based on the in vitro MRI studies, M1-MnO_2_ NMs with mesoporous flower-like morphology showed promising longitudinal relaxivity value (r_1_ = 5.73 mM^−1 ^s^−1^), with more efficient contrast enhancement effect compared to M2-MnO_2_ NMs having near-spherical morphology. The longitudinal relaxation rate of M1-MnO_2_ NMs was higher in the presence of MCF-7 cancer cells, which could be attributed to their more porous structure and high accessibility of water molecules to the Mn^+2^ paramagnetic centers. The obtained results revealed that by controlling the shape, size, and specific surface area of the MNMs as CA, the efficiency of MR imaging could be improved. *In vivo* studies on the synthesized flower-like M1-MnO_2_ NMs with dual-enzyme mimetic activities for targeted photodynamic cancer therapy will be our future purposes.

### Supplementary Information

Supplementary Information.

## Data Availability

The datasets used and/or analysed during the current study available from the corresponding author on reasonable request.
