# Supplementary material for: Tannic acid-mediated synthesis of flower-like mesoporous MnO2 nanostructures as T1–T2 dual-modal MRI contrast agents and dual-enzyme mimetic agents
Source: Sci Rep. 2023 Sep 5;13:14606. doi: 10.1038/s41598-023-41598-0 (PMC10480446; doi:10.1038/s41598-023-41598-0)
Supplement: Supplementary file 1 — Supplementary Information. [file 41598_2023_41598_MOESM1_ESM.docx]

Electronic Supplementary Information (ESI)

**Tannic Acid-Mediated Synthesis of flower-like mesoporous MnO_2_ Nanostructures as T_1_- T_2_ Dual-Modal MRI contrast agents and Dual-Enzyme Mimetic Agents**

Farzaneh Sorouri^a,b1^, Elham Gholibegloo^a1^, Tohid Mortezazadeh^c^, Sahar Kiani^b^, Alireza Foroumadi^d^, Loghman Firoozpour^d^, Mehdi Khoobi^a, e*^

*^a^ Department of Radiopharmacy, Faculty of Pharmacy, Tehran University of Medical Sciences, Tehran, Iran*

*^b^ Department of Brain and Cognitive Sciences, Cell Science Research Center, Royan Institute for Stem Cell Biology and Technology, ACECR, Tehran, Iran*

*^c^ Department of Medical Physics, School of Medicine, Tabriz University of Medical Sciences, Tabriz, Iran*

*^d^ Department of Medicinal Chemistry, Faculty of Pharmacy, Tehran University of Medical Sciences, Tehran, Iran*

*^e^ Biomaterials Group, The Institute of Pharmaceutical Sciences (TIPS), Pharmaceutical Sciences Research Center, Tehran University of Medical Sciences, 1417614411, Tehran, Iran*

^1^These authors have the same contribution to this paper.

*Corresponding author: m-khoobi@tums.ac.ir (mehdi.khoobi@gmail.com)


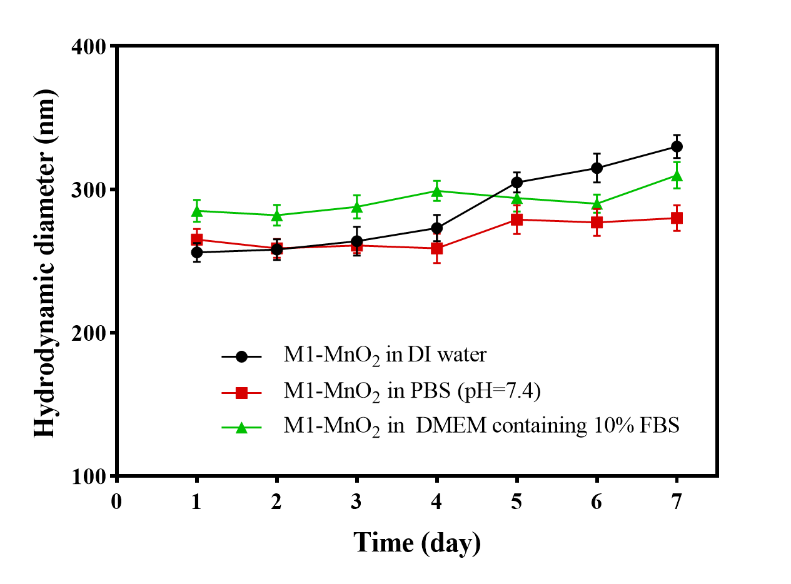


**Figure S1.** The stability of flower-like MnO_2_ NMs in DI water, PBS, and DMEM containing 10% FBS.


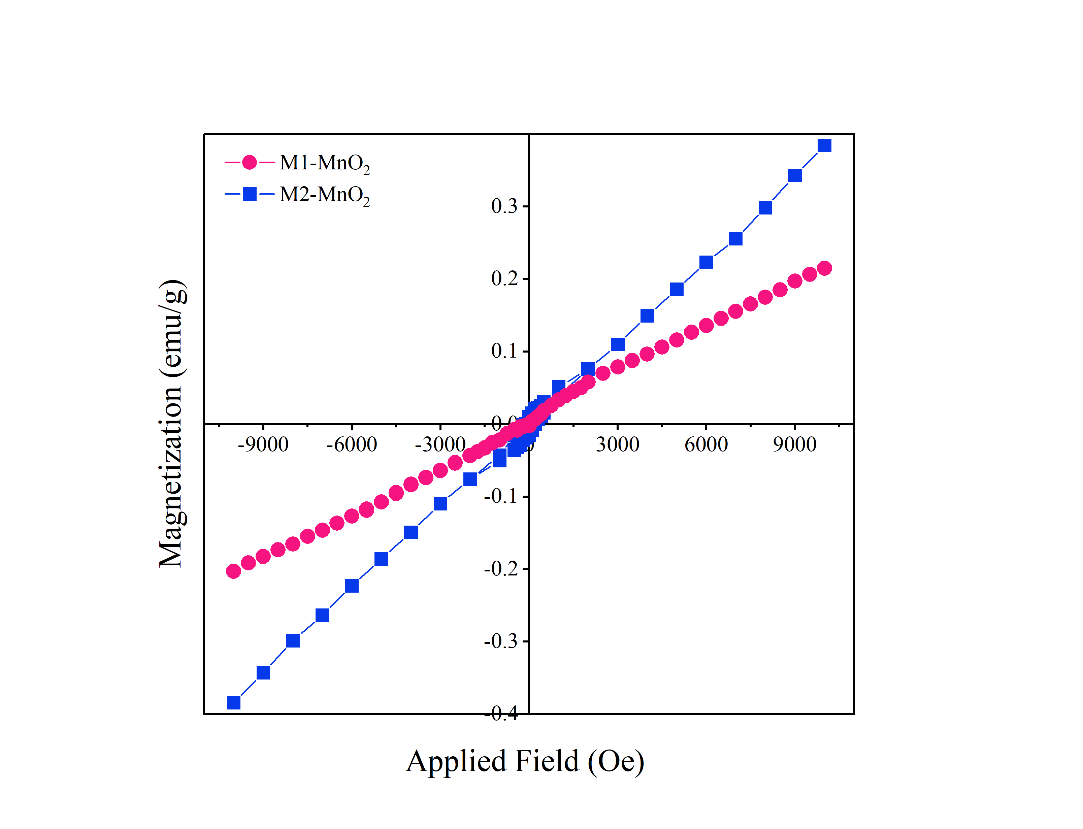


**Figure S2**. Magnetic hysteresis curves (M-H) of MNMs.


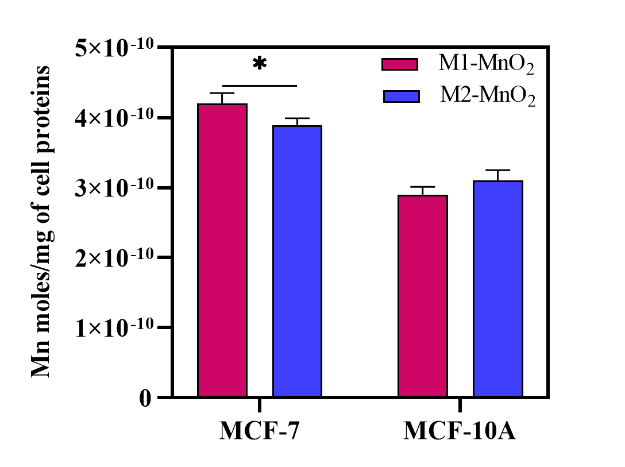


**Figure S3.** ICP-MS determination of the intracellular Mn content in MCF-7 and MCF-10A cells after incubation with M1-MnO_2_ and M2-MnO_2_ NMs.

Table S1. Comparison of the results of this study with previous studies reported in the literature related to MnO_2_-based nanosystems.

| **Agent** | **r_1_**  **(mM^-1^s^-1^)** | **r_2_** | **Morphology** | **Template** | **Nanozyme activity** | **Ref.** |
| --- | --- | --- | --- | --- | --- | --- |
| M1-MnO_2_ NMs | 5.73 | 5.49 | flower-like | TA | oxidase- like and peroxidase like activity | Current study |
| M2-MnO_2_ NMs | 5.49 | 3.78 | near-spherical | - |  | Current study |
| Ce6-DOX-MnO_2_ NPs | 6.43 | - | spherical | - | catalytic activity | ^1^ |
| PANI/PPy@Au@  MnO_2_ | 7.76 | - | yolk-shell | - | peroxidase-like activity | ^2^ |
| CNT@MnO_2_-PEG@Ce6 | 8.54 | - | hollow tubular | - | catalytic activity | ^3^ |
| Man-HA-MnO_2_ NPS | 7.96 | 45.05 | spherical | - | - | ^4^ |
| P-AgNCs-MnO_2_ | 8.23 | - | indistinguishable | - | - | ^5^ |
| MnO_2_ /HA nanosheets | 1.9 | - | sheet-like | - | - | ^6^ |
| MnO_2_–HA NPS | 1.6 | - | flower-like | - | - | ^7^ |
| BSA-MnO_2_ NPs | 4.76 | - | spherical | - | - | ^8^ |
| HMSNs@MnO_2_/apt | 9.25 | - | core/shell | silica | - | ^9^ |
| MS@MnO_2_ NPs | 6.91 | - | channel-like pores with MnO_2_ shell | - | - | ^10^ |
| MANPs-PTX NPS | 2.34 | - | spherical | - | - | ^11^ |
| MnO_2_-PEG-FA nanoshhets | 2.26 | - | sheet-like | - |  | ^12^ |
| MnO_2_@PtCo | - | - | flower like | - | oxidas like- activity | ^13^ |
| BSA-MnO_2_ NPs | - | - | - | BSA | oxidase- like and peroxidase like activity | ^14^ |

**References**

1 Hu, D. *et al.* Oxygen-generating hybrid polymeric nanoparticles with encapsulated doxorubicin and chlorin e6 for trimodal imaging-guided combined chemo-photodynamic therapy. *Theranostics* **8**, 1558 (2018).

2 Zhang, M. *et al.* A novel intelligent PANI/ PPy@Au@MnO2 yolk − shell nanozyme for MRI-guided ‘triple-mode’ synergistic targeted anti-tumor therapy. *J. Chem. Eng.* **424**, 130356 (2021).

3 Wang, D. *et al.* Retraction: A tumor-microenvironment fully responsive nano-platform for MRI-guided photodynamic and photothermal synergistic therapy. *J Mater Chem B* **10**, 9466-9467 (2022).

4 Song, M., Liu, T., Shi, C., Zhang, X. & Chen, X. Bioconjugated Manganese Dioxide Nanoparticles Enhance Chemotherapy Response by Priming Tumor-Associated Macrophages toward M1-like Phenotype and Attenuating Tumor Hypoxia. *ACS Nano* **10**, 633-647 (2016).

5 Yao, Y. *et al.* DNA-templated silver nanocluster/porphyrin/MnO2 platform for label-free intracellular Zn2+ imaging and fluorescence-/magnetic resonance imaging-guided photodynamic therapy. *ACS Appl. Mater. Interfaces* **11**, 13991-14003 (2019).

6 Hao, Y. *et al.* Manganese dioxide nanosheets-based redox/pH-responsive drug delivery system for cancer theranostic application. *Int. J. Nanomedicine.*, 1759-1778 (2016).

7 Zhao, L. *et al.* A nano-integrated diagnostic and therapeutic platform with oxidation–reduction reactions in tumor microenvironments. *Nanoscale Adv.* **2**, 2192-2202 (2020).

8 Zhang, M. *et al.* MnO2-based nanoplatform serves as drug vehicle and MRI contrast agent for cancer theranostics. *ACS Appl. Mater. Interfaces* **9**, 11337-11344 (2017).

9 Shi, Y., Guenneau, F., Wang, X., Hélary, C. & Coradin, T. MnO2-gated nanoplatforms with targeted controlled drug release and contrast-enhanced MRI properties: from 2D cell culture to 3D biomimetic hydrogels. *Nanotheranostics* **2**, 403 (2018).

10 Lin, L. S. *et al.* Simultaneous Fenton‐like ion delivery and glutathione depletion by MnO2‐based nanoagent to enhance chemodynamic therapy. *Angew. Chem.* **130**, 4996-5000 (2018).

11 Meng, L. *et al.* Facile deposition of manganese dioxide to albumin-bound paclitaxel nanoparticles for modulation of hypoxic tumor microenvironment to improve chemoradiation therapy. *Mol. Pharm.* **15**, 447-457 (2018).

12 Hao, Y. *et al.* Multifunctional nanosheets based on folic acid modified manganese oxide for tumor-targeting theranostic application. *Nanotechnology* **27**, 025101 (2015).

13 Wang, Z. *et al.* Biomimetic nanoflowers by self-assembly of nanozymes to induce intracellular oxidative damage against hypoxic tumors. *Nat. Commun.* **9**, 3334 (2018).

14 Liu, X. *et al.* BSA-templated MnO 2 nanoparticles as both peroxidase and oxidase mimics. *Analyst* **137**, 4552-4558 (2012).
